# Patients with Axial Spondyloarthritis Are at Risk of Developing Adhesive Capsulitis: Real-World Evidence Database Study in Taiwan

**DOI:** 10.3390/jcm9030787

**Published:** 2020-03-13

**Authors:** Shih-Wei Huang, Jr-Yi Wang, Che-Li Lin, Chi-Chang Huang, Tsan-Hon Liou, Hui-Wen Lin

**Affiliations:** 1 Department of Physical Medicine and Rehabilitation, Shuang Ho Hospital, Taipei Medical University, Taipei 23561, Taiwanpeter_liou@s.tmu.edu.tw (T.-H.L.); 2 Department of Physical Medicine and Rehabilitation, School of Medicine, College of Medicine, Taipei Medical University, Taipei 11031, Taiwan; 3 Graduate Institute of Sports Science, National Taiwan Sport University, Taoyuan 33301, Taiwan; john5523@ntsu.edu.tw; 4 Department of Orthopedic Surgery, Shuang Ho Hospital, Taipei Medical University, New Taipei City 23561, Taiwan; active9102078@gmail.com (J.-Y.W.); 11010@s.tmu.edu.tw (C.-L.L.); 5 Department of Orthopedics, School of Medicine, College of Medicine, Taipei Medical University, Taipei 11031, Taiwan; 6 Department of Mathematics, Soochow University, Taipei 11102, Taiwan; 7 Evidence-Based Medicine Center, Wan Fang Hospital, Taipei Medical University, Taipei 11696, Taiwan

**Keywords:** axial spondyloarthritis, ankylosing spondylitis, adhesive capsulitis, frozen shoulder, population-based study

## Abstract

Patients with axial spondyloarthritis (ax-SpA) present with inflammation invading the axial skeleton. Symptoms of ax-SpA interfere with patients’ quality of life, and peripheral symptoms are also noted. Human leukocyte antigen B27 was associated with adhesive capsulitis. However, epidemiological studies investigating the associated incidence and risk factors for patients with ax-SpA with adhesive capsulitis are limited. The data of patients with ax-SpA were recorded during the 2004–2008 period and followed to the end of 2010. The control cohort comprised age- and sex-matched non-ax-SpA subjects. A Cox multivariate proportional hazards model was applied to analyze the risk factors for adhesive capsulitis. The hazard ratio (HR) and adjusted hazard ratio (aHR) were estimated between the study and control cohorts after confounders were adjusted for. Effects of sulfasalazine (SSZ), methotrexate (MTX), and hydroxychloroquine (HCQ) use on adhesive capsulitis risk were also analyzed. We enrolled 2859 patients with ax-SpA in the study cohort and 11,436 control subjects. A higher incidence of adhesive capsulitis was revealed in the ax-SpA cohort: The crude HR was 1.63 (95% CI, 1.24–2.13; *p* < 0.001), and the aHR was 1.54 (95% CI, 1.16–2.05; *p* = 0.002). For patients with ax-SpA using SSZ or HCQ, no difference in aHR was noted compared with control participants, but patients with ax-SpA treated with MTX had higher HR and aHR than controls. Patients with ax-SpA are at risk for adhesive capsulitis. When these patients receive SSZ or HCQ, the risk of adhesive capsulitis can be lowered compared with that of the control cohort.

## 1. Introduction

Axial spondyloarthritis (ax-SpA) is a chronic inflammatory disease that mostly invades the axial skeleton. It comprises a number of inflammatory conditions related to the spine. In addition to ankylosing spondylitis (AS), types of spondyloarthritis (SpA) include reactive arthritis and psoriatic arthritis. When structural lesions are radiographically visualized on the spine or the sacroiliac joint, the patient can be classified as having AS. When no structural lesion is detected by radiographic imaging, the patient is classified as having nonradiographic ax-SpA. SpA usually starts in the second to third decade of life, and patients who are human leukocyte antigen B27 (HLA-B27)-positive generally are diagnosed 5 years earlier than patients who are HLA-B27-negative [1,2]. The prevalence of SpA (including peripheral forms) has been reported to be 0.20% in Southeast Asia and 1.61% in northern Arctic communities [3]. AS occurs more often among men than among women, with a ratio of approximately 2:1 to 3:1. However, no difference in gender distribution has been reported for patients with nonradiographic ax-SpA [2,4]. Patients with ax-SpA usually present with chronic back pain and stiffness over the pelvis and lower back. In addition, they can experience low back pain and stiffness that improve after exercise. The symptoms of ax-SpA often appear before age 40 years and can lead to work limitations and an increase in related medical costs to society [5,6].

Adhesive capsulitis, also called “frozen shoulder,” is a disease with symptoms of shoulder range-of-motion limitation and pain. It is usually characterized as idiopathic intense shoulder pain and subsequently fibrosis of the glenohumeral joint and contracture of the joint capsule occur [7]. It occurs in 2%–5% of the general population and is more predominant in women than in men [8]. Disability caused by symptoms of adhesive capsulitis can influence a patient’s work performance and increase medical costs [9]. Although many mechanisms of its pathogenesis have been hypothesized, such as inflammatory, immunological, and endocrinological mechanisms, the cause of adhesive capsulitis is still not well defined. Some studies have described associations of diabetes mellitus, thyroid disease, and gout with adhesive capsulitis [10,11,12]. Identification of a possible etiology is essential for further studies of the pathogenesis of adhesive capsulitis and to improve the quality of care of affected patients.

To our knowledge, the influence of ax-SpA on the development of adhesive capsulitis has not been investigated; furthermore, large-scale epidemiological studies of this association are lacking. Limited information is available regarding the risk of adhesive capsulitis in patients with ax-SpA. Therefore, we conducted a longitudinal, population-based study to investigate the risk of development of adhesive capsulitis in patients with ax-SpA.

## 2. Methods

### 2.1. Study Database

Using the International Classification of Diseases, Ninth Revision, Clinical Modification (ICD-9-CM), diagnosis code 720.0, we collected the data of patients with ax-SpA. After sex and age variables were controlled, the data of the comparison group, which included participants without ax-SpA diagnosis codes, were obtained from the same database. This retrospective longitudinal database study was conducted using the Longitudinal Health Insurance Database 2005 (LHID2005), which was previously maintained by the Taiwan National Health Insurance (NHI) Administration. The LHID2005 contains the claims data of 1 million beneficiaries, randomly sampled from among all the participants with registration of NHI coverage since 2005. Almost all the medial services provided by Taiwan’s NHI were included. LHID2005 contains the records of medical services such as clinic visits, medical prescriptions, intervention procedures, and surgery; inpatient diagnosis codes and basic demographic data of age and sex are also included. Although this database can provide information on the diagnostic evaluation services covered by Taiwan’s NHI, detailed outcomes and reports of examinations cannot be obtained from it. All the names and identity numbers were deidentified in LHID2005. This study was approved by the Institutional Review Board of the University of Taipei (UT-IRB No. IRB-2018-07). In addition, almost all the participants in study and control cohorts were Asians (Taiwanese).

### 2.2. Axial SpA Study Group Selection

**Figure 1:**
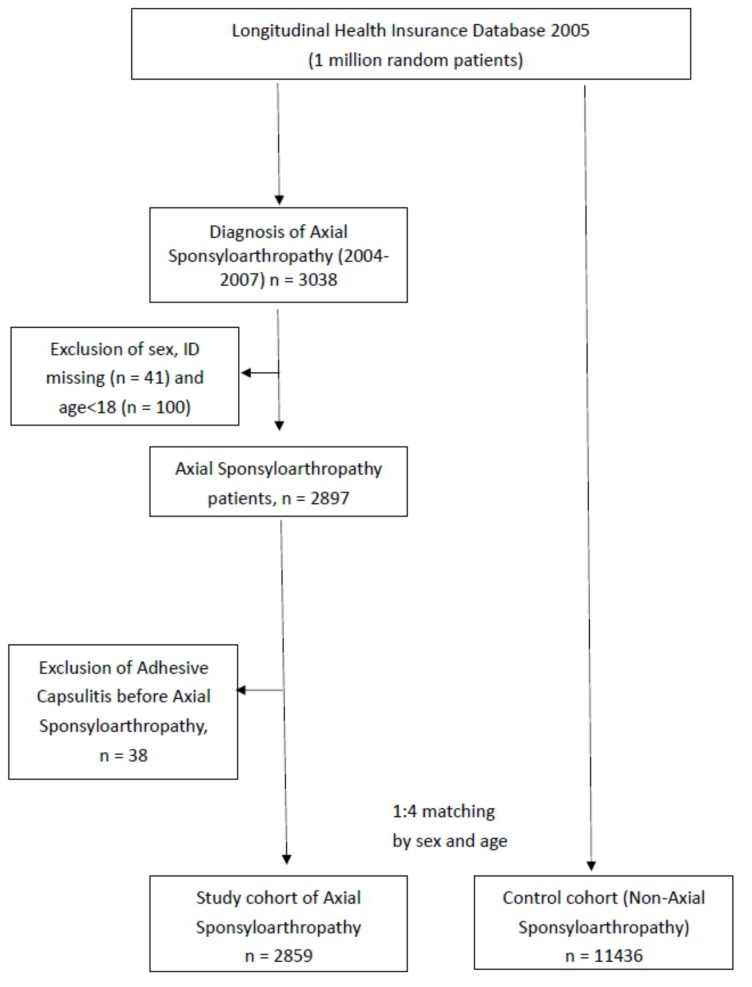
Flowchart showing the study design.

In the LHID2005, the diseases were recorded according to the ICD-9-CM diagnosis codes in the NHI database. We collected ax-SpA cohorts aged older than 18 years with an ICD-9-CM code of 720.0 from 2004 to 2007. The diagnosis of ax-SpA was based on diagnostic coding, which was determined by clinicians. To receive payment from the NHI system, clinicians follow the diagnostic rules and treatment guidelines for ax-SpA. Otherwise, professional physicians in related fields, such as rheumatology, will decline to pay the medical expenditures for ax-SpA. In addition, concerning the accuracy of coding, we collected only the data for at least two consecutive primary diagnosis codes for ax-SpA in the database. Patients with ax-SpA with previous adhesive capsulitis diagnosis codes before 2004, those with missing data, and those who died during the study period were excluded from the study cohort. Finally, 2859 patients with ax-SpA were collected in the study cohort. For comparison with the study cohort, we collected a control cohort, comprising participants who had no ax-SpA diagnosis codes, and matched participants with baseline variables of sex and age in a 1:4 ratio during the same time period as the study cohort (Figure 1).

### 2.3. Demographic Variables and Comorbidities

The demographic variables of age and sex; urbanization level; and medication usage of sulfasalazine (SSZ, which was the disease-modifying anti-rheumatic drug acting by the inhibition of prostaglandins and control of inflammation), methotrexate (MTX, which was known as a chemotherapy agent and immune system suppressant), and hydroxychloroquine (HCQ, which was known as used for the prevention and treatment of malaria and also used to treat patients with SLE or RA) were obtained from LHID2005 for analysis. Comorbidities of diabetes mellitus (ICD-9-CM codes 250 and 251), hypertension (ICD-9-CM codes 401–405), coronary heart disease (ICD-9-CM codes 410 and 412), thyroid disorders (ICD-9-CM codes 240–246), gout (ICD-9-CM code 274), and autoimmune disease (rheumatoid arthritis (RA) and systemic lupus erythematosus (SLE) with ICD-9-CM codes 714.0 and 710.0, respectively) were also recorded. The endpoint of follow-up was determined to be the occurrence of adhesive capsulitis (ICD-9-CM diagnosis code 726.0: adhesive capsulitis of shoulder) from the index date to the endpoint or until December 31, 2010, whichever was earlier, and the final-date observations were censored.

### 2.4. Statistical Analysis

We used Pearson’s chi-square test for analysis of demographic variables and comorbidities between the study and control cohorts. For analysis of risk and incidence of adhesive capsulitis between these two cohorts, we used the Cox model with adjustment for possible confounding factors such as comorbidities and demographics variables to analyze the crude hazard ratio (HR) and adjusted hazard ratio (aHR). In addition to the aforementioned variables, the effect of medication for ax-SpA, such as SSZ, MTX, and HCQ, and aHRs were analyzed in this model. Each dichotomous variable in the model was checked for proportionality by exploratory diagnostic log (-log(Survival)) plots in order to meet the proportional hazards assumption. All the dichotomous variables in the model were satisfied with the proportional hazards assumption. Also, the influence of the risk of adhesive capsulitis associated with use of these medications was analyzed and presented in Kaplan–Meier hazard curves for the study cohort during the follow-up period. All data analyses were performed using the Stata software package (version 11; StataCorp, College Station, TX, USA) and the SAS statistical software package (version 9.1.3; SAS Institute, Cary, NC, USA). A *P* value of <0.05 was considered statistically significant.

## 3. Results

In both cohorts, 82.5% of the patients were men, and the prevalence of comorbidities such as diabetes mellitus, chronic obstructive pulmonary disease, hypertension, hyperlipidemia, autoimmune disease, coronary heart disease, thyroid disease, and gout was higher in the ax-SpA cohort than in the control cohort (*p* < 0.001; Table 1).

The incidence of adhesive capsulitis was 263 per 100,000 person-years in the control cohort and 492 per 100,000 person-years in the ax-SpA cohort. For the risk of adhesive capsulitis compared with the control cohort, the crude hazard ratio (HR) was 1.63 (95% confidence interval (CI), 1.24–2.13; *p* < 0.001), and the aHR was 1.54 (95% CI, 1.16–2.05; *p* = 0.002; Table 2).

**Figure 2:**
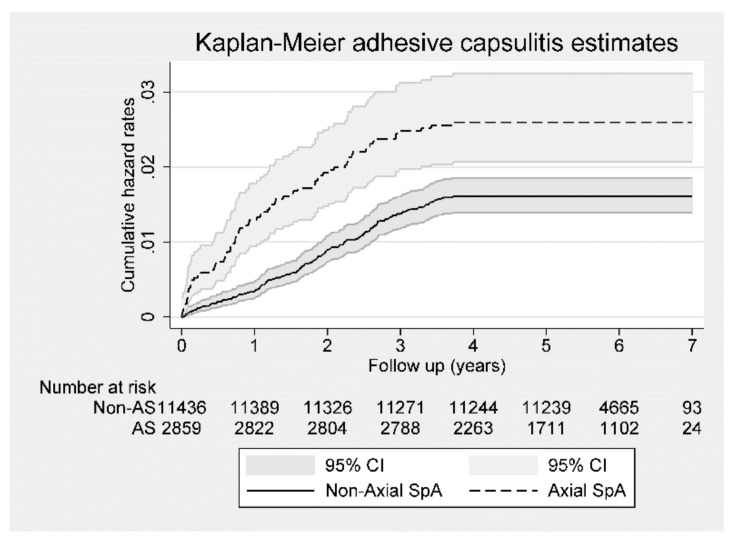
Kaplan–Meier hazard curve for adhesive capsulitis in patients with axial spondyloarthritis (Axial SpA) and control subjects for the 7-year follow-up period.

Figure 2 presents the Kaplan–Meier hazard curves for the risk of adhesive capsulitis in the ax-SpA and control cohorts during the 7-year follow-up period. A log-rank analysis revealed that the patients in the gout cohort had higher HRs (*p* < 0.001) than those in the control cohort.

In the ax-SpA cohort without SSZ medication compared with the control cohort, the crude HR was 1.71 (95% CI, 1.30–2.26; *p* < 0.001), and the aHR was 1.57 (95% CI, 1.18–2.08; *p* < 0.01). However, adhesive capsulitis risk between the control cohort and patients with ax-SpA who received SSZ medication was not statistically different (Table 3).

**Figure 3:**
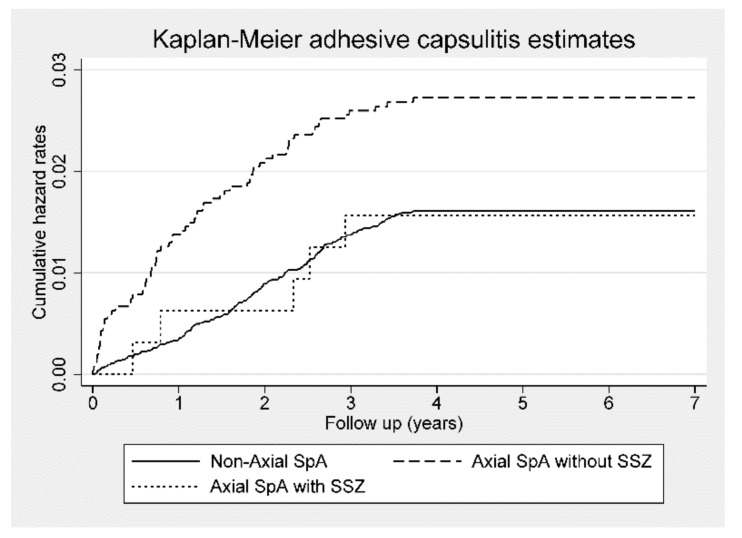
Kaplan–Meier hazard curve for adhesive capsulitis in patients with axial spondyloarthritis (Axial SpA) with or without sulfasalazine (SSZ) use and control subjects over the 7-year follow-up period.

Figure 3 presents the Kaplan–Meier hazard curves for the risk of adhesive capsulitis among patients with ax-SpA not receiving SSZ, patients with SpA treated with SSZ, and control subjects during the 7-year follow-up period.

**Figure 4:**
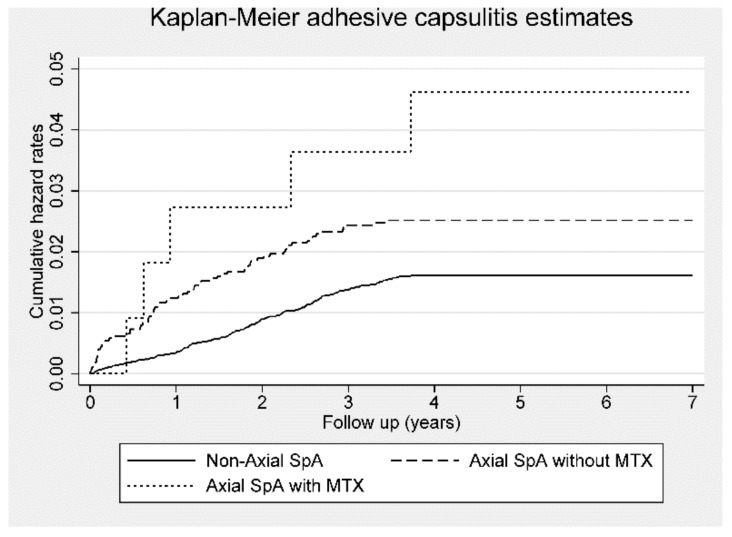
Kaplan–Meier hazard curve for adhesive capsulitis in patients with axial spondyloarthritis (Axial SpA) with or without methotrexate (MTX) use and control subjects over the 7-year follow-up period.

The crude HR and aHR for risk of adhesive capsulitis in the patients with ax-SpA without MTX treatment were 1.58 (95% CI, 1.20–2.08; *p* < 0.01) and 1.51 (95% CI, 1.13–2.00; *p* < 0.01), respectively, during the 7-year follow-up period. Patients with ax-SpA treated with MTX had a crude HR of 2.87 (95% CI, 1.18–7.0; *p* < 0.05) and an aHR of 3.01 (95% CI, 1.21–7.49; *p* < 0.05) (Table 3). Figure 4 presents Kaplan–Meier hazard curves showing that patients with ax-SpA treated with MTX had a higher risk of adhesive capsulitis than those not receiving MTX treatment and control participants during the 7-year follow-up period.

**Figure 5:**
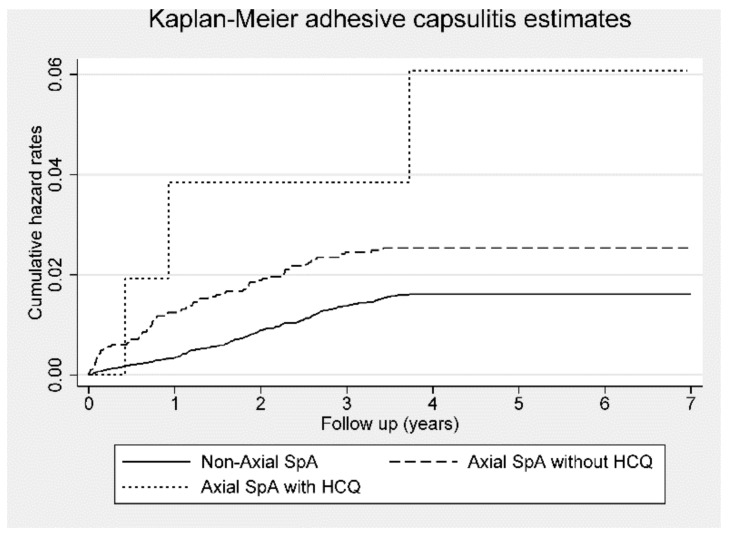
Kaplan–Meier hazard curve for adhesive capsulitis in patients with axial spondyloarthritis (Axial SpA) with or without hydroxychloroquine (HCQ) use and control subjects over the 7-year follow-up period.

Patients with ax-SpA without HCQ treatment had a significantly higher risk of adhesive capsulitis, with a crude HR of 1.59 (95% CI, 1.21–2.09; *p* < 0.01) and aHR of 1.53 (95% CI, 1.16–2.02; *p* < 0.01). Although patients with ax-SpA treated with HCQ had a crude HR of 3.69 (95% CI, 1.17–11.54; *p* < 0.05) for adhesive capsulitis, the aHR was not significantly different between control participants and patients with ax-SpA receiving HCQ (Table 3). Figure 5 displays the trends of Kaplan–Meier hazard curves for the risk of adhesive capsulitis among patients with ax-SpA with or without HCQ treatment and control subjects during the follow-up period.

## 4. Discussion

Until now, no relevant study had been conducted on the risk of adhesive capsulitis among patients with ax-SpA. Our large-scale, longitudinal, real-world data analysis found that patients with ax-SpA had a higher risk of adhesive capsulitis. When patients with ax-SpA were treated with SSZ or HCQ, their risk of adhesive capsulitis was not different from that of the control cohort. However, among patients with ax-SpA who did not receive these medications, the risk of adhesive capsulitis was higher than that in the control cohort. In contrast to use of SSZ and HCQ, patients with ax-SpA treated with MTX had a higher risk of adhesive capsulitis than the control cohort. The finding of the different influence of medication on the risk of adhesive capsulitis among patients with ax-SpA could help to identify the potential pathogenesis of capsulitis.

The pathogenesis of adhesive capsulitis remains under investigation. As previously stated, at the onset of adhesive capsulitis, inflammation of the glenohumeral synovium can occur before the biological cascade leading to fibrosis, which can result in limited range of motion of the shoulder [13]. Regarding the inflammatory cascade, the synovial cells and fibroblasts of the shoulder capsule were targets of inflammatory invasion [14]. The inflammatory cascade is related to fibroblastic hyperplasia induced by cytokines such as transforming growth factor-β, tumor necrosis factor-α, platelet-derived growth factor, interleukin 1 (IL-1), and IL-6 [15,16]. This can also be confirmed by observing the histologic presentation of fibroblast cell proliferation with increased matrix metalloproteinase and fibrogenic growth factors in patients with adhesive capsulitis [17].

In addition to the involvement of the inflammatory cascade in the pathogenesis of idiopathic adhesive capsulitis, genetics plays a role. A previous study found that a higher percentage of patients with primary adhesive capsulitis were white (76%) than black (24%) [18]. Regarding familial clustering, previous studies found a positive family history in first-degree relatives associated with adhesive capsulitis [19]. Authors of a recent systematic review investigated the association of adhesive capsulitis with family history and race by collecting twin and familial clustering studies; they found a genetic association in adhesive capsulitis [20]. In the same review, the authors also investigated HLA-B27 and the risk of adhesive capsulitis through a meta-analysis. They found a significantly higher rate of HLA-B27 positivity among patients with idiopathic adhesive capsulitis than in controls [20]. HLA-B27 is a protein involved in inflammatory responses, and patients with ax-SpA have a high HLA-B27-positive rate. HLA-B27 may play an important role in explaining the risk of developing adhesive capsulitis among patients with ax-SpA.

Among patients with ax-SpA treated with SSZ, the HR and aHR were not different from those in the control cohort. As aforementioned, the cornerstone of understanding adhesive capsulitis is the inflammatory cascade; therefore, we hypothesized that the risk of adhesive capsulitis could be decreased by controlling the inflammatory process with SSZ. On the basis of the current recommendations for the management of ax-SpA, SSZ was considered as an option for peripheral involvement in patients with ax-SpA [21,22]. In addition to the effects of SSZ in patients with ax-SpA with peripheral symptoms, the Cochrane review by Chen et al. suggested that patients with ax-SpA with increased erythrocyte sedimentation rate and shorter disease duration could benefit from SSZ treatment [22]. With regard to MTX, our study showed that patients with ax-SpA were still at risk for adhesive capsulitis even with the use of MTX. MTX was considered an effective treatment for RA [23,24]. However, the effect of MTX in patients with ax-SpA is still not well recognized. Haibel et al. investigated the use of a higher dose of MTX for patients with AS with axial involvement and found that it was not effective for treating patients with AS [25]. Therefore, despite being a disease-modifying anti-rheumatic drug (DMARD), MTX is not effective for controlling the inflammatory process of ax-SpA and does not lessen the risk of adhesive capsulitis. Our data even showed a higher risk of adhesive capsulitis among patients with ax-SpA. However, no mechanism of pathogenesis to explain this result was found. There was another potential explanation that MTX is selected to treat higher severity of ax-SpA patients, but only limited information on disease severity can be obtained from the LHID2005 database. Moreover, for instance, it has been observed that SSZ has a dual role in decreasing the production of TNF-alpha and prostaglandins as well as increasing adenosine production, and both effects may be in agreement with the possible protective action of SSZ [26,27,28]. SSZ and MTX partially share the same mechanism of action, although SSZ may be considered a more multifaceted molecule [26,29]. So this might point toward a possible reason for the different risk modulation demonstrated by the two drugs. They may have a partially different therapeutic mechanism of action and lead opposite roles of adhesive capsulitis risk among ax-SpA patients. HCQ is known as a medication for malaria and is also used to treat patients with SLE or RA. Our study revealed a higher risk of adhesive capsulitis with HCQ based on a crude HR for patients with ax-SpA. However, no statistically significant difference in aHR was observed compared with the control cohort when other comorbidities were controlled for among patients with ax-SpA treated with HCQ. The explanation for this could be that HCQ can inhibit the development of insulin resistance and control diabetes mellitus among patients with autoimmune diseases [30,31]. Diabetes is a well-known risk factor for adhesive capsulitis, and we hypothesized that HCQ could lower the risk of adhesive capsulitis by controlling diabetes mellitus among patients with ax-SpA. Further studies are required to clarify possible mechanisms to explain the changes in the risk of adhesive capsulitis when HCQ is administered for patients with ax-SpA.

Our study demonstrated that patients with ax-SpA were at higher risk of adhesive capsulitis. When these patients used SSZ, no difference in adhesive capsulitis was observed compared with the control cohort. Regarding comorbidities such as diabetes mellitus, use of HCQ in patients with ax-SpA was not associated with a statistically significant difference in adhesive capsulitis risk compared with the control cohort.

The strengths of this study are its large sample size and the fact that it is the first epidemiological study to investigate the risk of adhesive capsulitis among patients with ax-SpA treated with SSZ, MTX, or HCQ. Nevertheless, some limitations should be addressed. First, the diagnosis of ax-SpA, adhesive capsulitis, and related comorbidities was based on ICD-9-CM codes in LHID2005. To ensure proper payment for treatments and evaluation of these diseases, the NHI Bureau regularly reviews medical records and checks that management is compatible with the diagnostic coding. We also enrolled only two consecutive coding cases in the study cohort to eliminate incorrect coding. Second, other demographic variables, such as cigarette smoking, alcohol consumption, employment, and exercise of leisure, cannot be obtained from the database. Although 20%–30% of patients with adhesive capsulitis reported a history of minor trauma to the shoulder, limited evidence exists to prove that adhesive capsulitis is a posttraumatic condition [32,33]. Third, the severity of ax-SpA cannot be stratified because the laboratory data cannot be accessed using LHID2005. The positive rate of HLA-B27, an inflammatory marker involving joints, range of motion, and radiographic information, was lacking. Finally, the onset of ax-SpA was determined by diagnostic coding, and the definite time period of disease onset cannot be known unless patients with ax-SpA visited a doctor for their symptoms. Despite these limitations, our study provides crucial information showing that patients with ax-SpA were at risk of adhesive capsulitis and that the risk was not different from that in the control cohort with use of SSZ or HCQ.

## 5. Conclusions

Our longitudinal population-based cohort study results showed that patients with ax-SpA were at higher risk of adhesive capsulitis than patients without this condition. Further analysis revealed no statistically significant difference in aHR between patients with ax-SpA treated with SSZ or MCQ and the control cohort with regard to adhesive capsulitis risk. We hypothesized that HLA-B27 and the inflammatory cascade are important contributing factors in adhesive capsulitis in patients with ax-SpA. To decrease the risk of adhesive capsulitis, proper use of DMARDs such as SSZ or MCQ is recommended for patients with ax-SpA. Further studies to investigate the detailed mechanism of this phenomenon are recommended.

## Figures and Tables

**Table 1:** Baseline demographic characteristics and comorbidities for age- and sex-matched patients in the ankylosing spondylitis and non-ankylosing spondylitis cohorts (*n* = 14,295).

Baseline Variable	Patients with ax-SpA (*n* = 2859)		Patients without ax-SpA (*n* = 11,436)		*p* Value
	No.	(%)	No.	(%)	
Characteristics					
Age (y)					1.00
18–30	729	25.5	2916	25.5	
31–40	679	23.7	2716	23.7	
41–50	613	21.4	2452	21.4	
51–60	408	14.3	1632	14.3	
61–70	237	8.3	948	8.3	
>70	193	6.8	772	6.8	
Sex					1.00
Male	1828	63.9	7312	63.9	
Female	1031	36.1	4124	36.1	
**Comorbid Medical Disorders**					
DM					0.007
Yes	350	12.2	1200	10.5	
No	2509	87.8	10,236	89.5	
Coronary heart disease					<0.001
Yes	377	13.2	1086	9.5	
No	2482	86.8	10,350	90.5	
Hypertension					<0.001
Yes	722	25.3	2518	22.0	
No	2137	74.7	8918	78.0	
COPD					<0.001
Yes	634	22.2	1917	16.8	
No	2225	77.8	9519	83.2	
Hyperlipidemia					<0.001
Yes	557	19.5	1793	15.7	
No	2302	80.5	9643	84.3	
Autoimmune disease (RA, SLE)					<0.001
Yes	304	10.6	302	2.6	
No	2555	89.4	11,134	97.4	
Thyroid disease					<0.001
Yes	186	6.5	451	3.9	
No	2673	93.5	10,985	96.1	
Gout					<0.001
Yes	395	13.8	1199	10.5	
No	2464	86.2	10,237	89.5	
Medication therapy					
Sulfasalazine					<0.001
Yes	319	11.2	18	0.2	
No	2540	88.8	11,418	99.8	
Methotrexate					<0.001
Yes	110	3.8	28	0.2	
No	2749	96.2	11,408	99.8	
Hydroxychloroquine					<0.001
Yes	52	1.8	31	0.3	
No	2807	98.2	11,405	99.7	

Ax-SpA, axial spondyloarthritis; COPD, chronic obstructive pulmonary disease; DM, diabetes mellitus; RA, rheumatoid arthritis; SLE, systemic lupus erythematosus.

**Table 2:** Incidence and hazard ratio for adhesive capsulitis between patients with and without axial spondyloarthritis during the 7-year follow-up (*n* = 14,295).

Presence of Adhesive Capsulitis	Patients without ax-SpA	Patients with ax-SpA
Follow-up period		
Yes/total	184/2589	74
Person-years	69,853	15,040
Incidence per 100,000 person-years	263	492
Crude hazard ratio (95% CI)	1.00	1.63 *** (1.24–2.13)
Adjusted hazard ratio (95% CI)	1.00	1.54 *** (1.16–2.05)

Ax-SpA, axial spondyloarthritis; CI, confidence interval. Adjusted for autoimmune disease (rheumatoid arthritis, systemic lupus erythematosus), diabetes mellitus, hypertension, hyperlipidemia, coronary heart disease, thyroid disease, gout, chronic obstructive pulmonary disease, and sulfasalazine (SSZ), methotrexate (MTX), and hydroxychloroquine (HCQ) medication therapy. *** *p* < 0.001.

**Table 3:** Incidence, crude and adjusted hazard ratios, and 95% confidence intervals for adhesive capsulitis during the 7 years of follow-up (*n* = 14,295).

Presence of FS	Non-ax-SpA	Patients with ax-SpA	
		Without Sulfasalazine	With Sulfasalazine
Crude HR (95% CI)	1.00	1.71 *** (1.30–2.26)	0.97 (0.40–2.37)
Adjusted HR (95% CI)	1.00	1.57 ** (1.19–2.08)	1.32 (0.53–3.25)
		Without methotrexate	With methotrexate
Crude HR (95% CI)	1.00	1.58 ** (1.20–2.08)	2.87 * (1.18–7.0)
Adjusted HR (95% CI)	1.00	1.51 ** (1.14–2.00)	3.01 * (1.21–7.49)
		Without hydroxychloroquine	With hydroxychloroquine
Crude HR (95% CI)	1.00	1.59 ** (1.21–2.09)	3.69 * (1.17–11.54)
Adjusted HR (95% CI)	1.00	1.53 ** (1.16–2.03)	2.51 (0.77–8.10)

Ax-SpA, axial spondyloarthritis; CI, confidence interval. Adjusted for patient’s age, sex, autoimmune disease, diabetes mellitus, hypertension, hyperlipidemia, coronary heart disease, thyroid disease, gout, and chronic obstructive pulmonary disease. *** *p* < 0.001; ** *p* < 0.01; * *p* < 0.05.
